# Hypoxia-induced transcription of dopamine D3 and D4 receptors in human neuroblastoma and astrocytoma cells

**DOI:** 10.1186/1471-2202-10-92

**Published:** 2009-08-04

**Authors:** Melinda Bence, Eva Kereszturi, Viktor Mozes, Maria Sasvari-Szekely, Gergely Keszler

**Affiliations:** 1 Department of Medical Chemistry, Molecular Biology and Pathobiochemistry, Semmelweis University, POB 260, H-1444 Budapest, Hungary

## Abstract

**Background:**

Dopaminergic pathways that influence mood and behaviour are severely affected in cerebral hypoxia. In contrast, hypoxia promotes the differentiation of dopaminergic neurons. In order to clarify the hypoxic sensitivity of key dopaminergic genes, we aimed to study their transcriptional regulation in the context of neuroblastoma and astrocytoma cell lines exposed to 1% hypoxia.

**Results:**

Quantitative RT-PCR assays revealed that the transcription of both type D3 and D4 postsynaptic dopamine receptors (DRD3 and DRD4) was induced several fold upon 2-day hypoxia in a cell-specific manner, while the vascular endothelial growth factor gene was activated after 3-hr incubation in hypoxia. On the other hand, mRNA levels of type 2 dopamine receptor, dopamine transporter, monoamino oxidase and catechol-O-methyltransferase were unaltered, while those of the dopamine receptor regulating factor (DRRF) were decreased by hypoxia. Notably, 2-day hypoxia did not result in elevation of protein levels of DRD3 and DRD4.

**Conclusion:**

In light of the relatively delayed transcriptional activation of the DRD3 and DRD4 genes, we propose that slow-reacting hypoxia sensitive transcription factors might be involved in the transactivation of DRD3 and DRD4 promoters in hypoxia.

## Background

The brain is considered a fully aerobic organ as it requires about 20% of total oxygen consumption in humans [1]. Interruption of steady oxygen supply results in focal necrosis and causes severe dysfunction in the ischemic penumbra [2]. Numerous studies underlined the seminal role of hypoxia inducible factor-1α (HIF-1α) in governing the hypoxic response in both neurons and glial cells [3,4]. The neuroprotective role of HIF-1α has been demonstrated in the ischemic penumbra through erythropoietin induction [5] as well as in mediating a neuroprotective response to amyloid-β peptide [6]. However, the regulation of central neurotransmission systems has not been thoroughly investigated under hypoxic conditions, although their inadequate adaptation might contribute to the development of cerebral palsy and abnormal behavioural patterns in patients affected by pre- or postnatal cerebral hypoxia, respectively [7-9].

Apart from its well-known functions in the nigro-striatal pathway, dopamine plays a very important role in the regulation of mood, affections, impulsivity and cognitive functions in the limbic system [10]. Dopaminergic neurotransmissison has been shown to be exquisitely vulnerable to ischemic-anoxic insults, and hypoxic derangements of the dopamine system have been implicated in the pathogenesis of cerebral palsy, schizophrenia and minimal brain dysfunction such as attention deficit hyperactivity disorder (ADHD) [11,12]. On the other hand, hypoxia has been implicated in promoting differentiation of neuronal precursor cells to dopaminergic neurons through activation of HIF-1α [13,14].

Our current understanding of dopaminergic signalling in hypoxia is further confounded by results of recent *in vivo *studies showing that hypoxic regulation of key dopaminergic genes is highly tissue-specific, and strongly influenced by the duration of hypoxic periods. Among these factors, most of attention has been attributed to the dopamine D2 receptor (DRD2) due to its pathological role in schizophrenia. DRD2 mRNA levels show an early and transient reduction in the striatum after hypoxia-ischemia in newborn rats [15], and attenuation of DRD2 mediated inhibition of calcium influx in pheochromocytoma cells has been reported in hypoxia [16]. On the other hand, Huey and Powell revealed that hypoxia modulates DRD2 expression in a tissue-dependent manner [17]. For instance, DRD2 mRNA levels initially increased in the caudal nucleus tractus solitarius in rats in response to hypoxia, but then significantly decreased after 48 h (and longer) hypoxic treatment. A similar tendency was unveiled in the rat carotid body, too. In contrast, hypoxia profoundly increased DRD2 mRNA in the rostral nucleus tractus solitarius at all time points investigated [17]. A study conducted on rabbit brains also revealed that hypoxic expression patterns of DRD1 and DRD2 in different brain areas are far from being uniform [18]. Moreover, widely accepted concepts like induction of the tyrosine hydroxylase gene by hypoxia [19] have been challenged by recent studies finding practically unaltered or slightly decreased transcript and protein levels upon hypoxia [20,21]. To our best knowledge, however, the hypoxic modulation of DRD3 and DRD4 receptors, two highly analyzed polymorphic determinants of psychiatric disorders [22-24], has not been addressed yet experimentally.

Previously we studied the functional effects of DRD4 promoter polymorphisms on gene expression [25], and reinforced the molecular function of a promoter variant characterized earlier [26]. In the present study, we aimed to investigate the transcriptional regulation of a set of dopamine-specific genes by measuring their mRNA and protein levels upon short-term hypoxic treatment of a neural (SK-NF-I) and a glial (CCF-STTG1) cell line. These cell lines were chosen since they correspond to the main cell types of the brain, neurons and astrocytes; moreover, both of them express DRD2, DRD3 and DRD4 receptors. We found that the transcription of the type D3 and D4 postsynaptic dopamine receptors (DRD3 and DRD4) was induced several fold upon 2-day hypoxia in a cell-specific manner.

## Results

### Expression and transcriptional activity of HIF-1α in SK-NF-I and CCF-STTG1 cells

**Figure 1:**
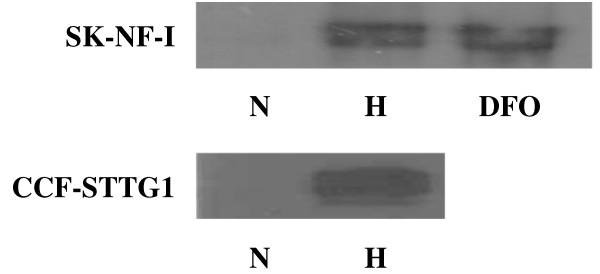
**Expression of HIF-1α in SK-NF-I and CCF-STTG1 cells**. Parallel cultures were incubated in normoxia (21% O_2_, lane "N") or in hypoxia (1% O_2_, lane "H") for 8 hours, or were treated with 100 μM desferrioxamine for 8 hours in normoxic atmosphere (lane "DFO") where indicated. Cells were subsequently harvested, extracted and samples were resolved by SDS-PAGE, blotted and probed with an anti-HIF-1α antibody at a dilution of 1:1000. Three blots were made from 3 independent biological samples and a representative image is shown.

Hypoxia-dependent transcriptional activation of genes is mostly governed by HIF-1α. This fact prompted us to analyze the expression patterns of HIF-1α in the SK-NF-I human neuroblastoma and CCF-STTG1 human astrocytoma cell line. To this end, cells were challenged either with 1% hypoxia or with 100 μM desferrioxamine (DFO), a hypoxia-mimicking agent that is known to stabilize HIF-1α through blocking the activity of proline hydroxylases [27]. As it can be seen in Fig. 1, the HIF-1α protein was undetectable in normoxic cultures, while both short-term hypoxic or DFO treatment dramatically upregulated the protein levels of this hypoxia dependent transcription factor. Importantly, two separate bands were recognized by the specific HIF-1α antibody that might correspond to differentially spliced variants [28]. In neuroblastoma cells, both bands were induced equally, while in CCF-STTG1 cells the staining of the upper band was more pronounced (Fig. 1).

**Figure 2:**
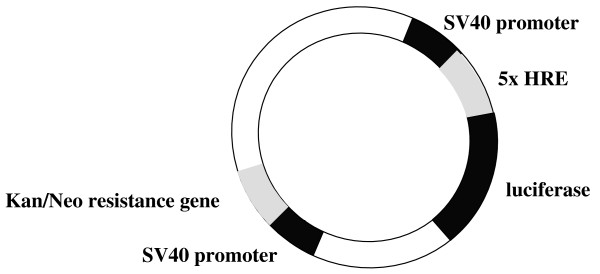
**Schematic map of the pSV40-5×HRE-luc reporter construct**.

**Figure 3:**
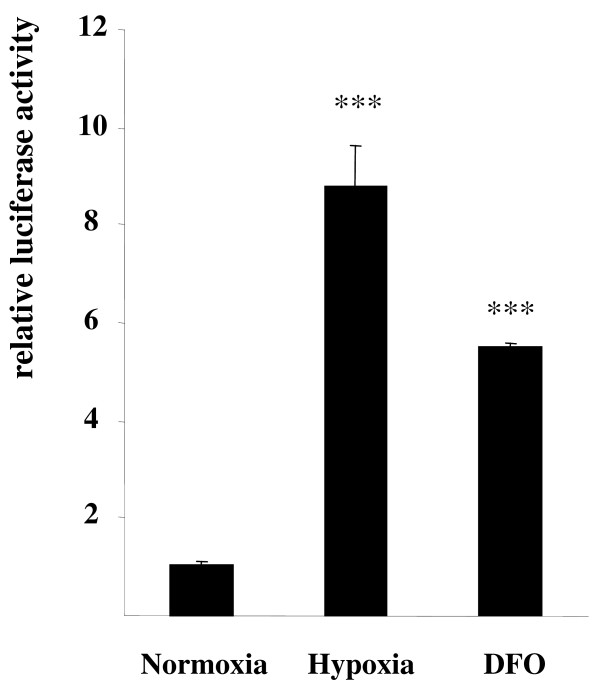
**Enhancement of HIF-1α activity in SK-NF-I cells upon hypoxia or DFO treatment**. SK-NF-I cells were transiently transfected with the pSV40-5×HRE-luc reporter construct (Fig. 2) and incubated under normoxic or hypoxic conditions, or treated with 100 μM DFO, respectively, for 24 hours. Luciferase activities normalized to β-galactosidase levels are displayed as fold increments over the control activity. Data are means of three parallel determinations from three independent experiments (N = 9); bars represent standard deviations (*: p < 0.05; **: p < 0.01 and ***: p < 0.001).

In order to directly monitor the transcriptional activity of HIF-1α in neuroblastoma cells, we generated a luciferase reporter vector containing multiple hypoxia responsive elements inserted in the SV40 strong promoter (Fig. 2). This construct was transiently transfected into SK-NF-I cells which were subsequently subjected to 1% hypoxia or treated with DFO as described in Methods. Notably, both hypoxia and DFO treatment enhanced the activity of this reporter construct several fold over basal levels (Fig. 3). These results proved that the hypoxic signalling pathway is intact and readily inducible in SK-NF-I cells.

### Induction of dopamine receptors 3 and 4 upon hypoxia

We aimed to study the expression of a set of dopaminergic neurotransmission specific genes under hypoxia in the SK-NF-I and astrocytoma model system. To this end, parallel cultures were maintained both in hypoxia and normoxia, and the temporal pattern of gene expression was followed by quantitative reverse transcription PCR using gene-specific TaqMan probes.

Finding a stable endogenous control gene is the cornerstone of the validation of qRT-PCR data. In order to select an optimal hypoxia-insensitive reference gene, we sought to screen for amplification efficiency and overall stability the mRNA levels of the following five candidate genes widely used in qRT-PCR studies: β-actin, hydroxymethylbilane synthase (HMBS), hypoxanthine guanine phosphorybosyltransferase (HPRT), P0 large ribosomal protein (RPLP0) and RNA polymerase II (RPII).

Table 1 shows the representative relative expression levels of these potential internal control genes measured in 8-hr hypoxic samples in SK-NF-I cells. Highly similar results were obtained in astrocytoma cells (data not presented). Based on these data, three reliable control pairs have been found (HPRT-RPLP0, HPRT-RPII and RPLP0-RPII), whereat the relative expression ratios were closest to 1. Of them, we chose the RPLP0 gene as internal control.

**Table 1:** Selection of the optimal internal control gene for qRT-PCR assays in SK-NF-I cells.

	RPLP0	β-actin	RPII	HMBS
**HGPRT**	1.04	2.83	1.17	2.49
**HMBS**	2.60	1.14	2.13	
**RPII**	1.22	2.42		
**β-actin**	2.97			

Data indicate the ratios of 2^-ΔCT ^values of corresponding gene pairs. The ratios were calculated by dividing the greater value with the smaller one so as to get quotients exceeding 1. C_T _values were determined by calculating the means of three parallel PCR amplifications from three independent cDNA samples (N = 9) prepared from SK-NF-I cultures kept for 8 hrs in 1% hypoxia.

**Figure 4:**
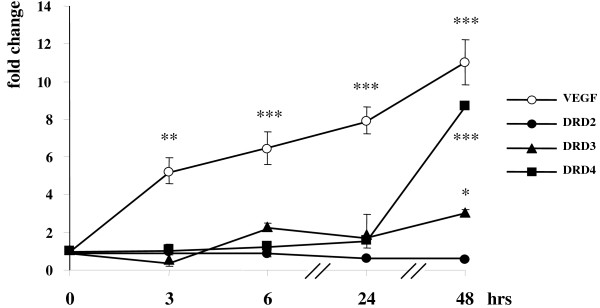
**Induction of dopamine D3 and D4 receptor mRNA levels in SK-NF-I cells upon hypoxia**. Parallel cultures of SK-NF-I cells were maintained under normoxic or hypoxic conditions for the indicated time periods. Cells were harvested, total mRNA was isolated and analyzed in real-time reverse transcription PCR assays with TaqMan probes specific for the human DRD2 (circles), DRD3 (triangles) and DRD4 (squares) receptors. VEGF (filled circles) was included as a well-known control target gene of HIF-1α. Expression levels were normalized to the RPLP0 internal control gene. All data are expressed as fold changes relative to levels measured in parallel normoxic samples. Data are means of three parallel determinations from three independent experiments (N = 9); bars represent standard deviations (*: p < 0.05; **: p < 0.01 and ***: p < 0.001).

**Figure 5:**
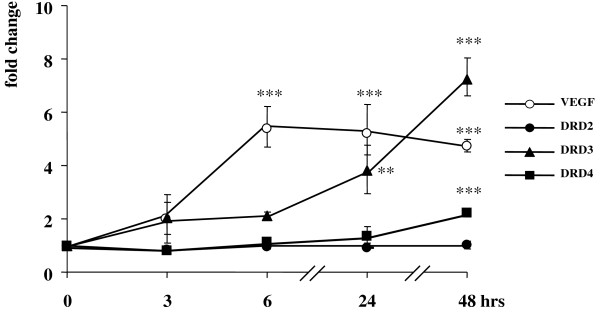
**Induction of dopamine D3 and D4 receptor mRNA levels in CCF-STTG1 cells upon hypoxia**. Parallel cultures of CCF-STTG1 cells were kept, treated and analyzed as described in Fig. 4. Data are means of three parallel determinations from three independent experiments (N = 9); bars represent standard deviations (*: p < 0.05; **: p < 0.01 and ***: p < 0.001).

No significant changes in the mRNA levels of dopamine D2, D3 and D4 receptors (DRD2, DRD3 and DRD4), the dopamine transporter (DAT), monoamino oxidase A (MAOA), catechol-O-methyltransferase (COMT) and vascular endothelial growth factor (VEGF) were revealed in samples kept for 0–48 hrs in normoxia (data not shown). On the contrary, both DRD3 and DRD4 receptor mRNA levels were upregulated upon long-term (48 hrs) incubation of SK-NF-I and CCF-STTG1 cells in 1% hypoxia (Figs. 4 and 5). In neuroblastoma cells, the induction of DRD4 was much more pronounced (about eightfold compared to 48-hr untreated control levels) than that of DRD3 (Fig. 4). In contrast, the DRD3 was much more inducible than DRD4 in astrocytoma cells, and its mRNA levels were highly elevated already after 24 hr hypoxia (Fig. 5). However, the induction pattern of both receptors differed profoundly from that of VEGF, a positive control gene known to be directly activated by HIF-1α: transcription of the VEGF gene was induced already after 3 hr hypoxia and its mRNA levels dynamically increased over the entire incubation period in both cell lines.

Importantly, the DRD2 gene did not prove to be hypoxia sensitive at all in either cell lines (Figs. 4 and 5). Furthermore, no remarkable alterations were revealed in the mRNA levels of COMT, MAOA and DAT upon hypoxia in either cell lines investigated (Table 2).

**Table 2:** Relative mRNA levels of catechol-O-methyltransferase (COMT), monoamino oxidase A (MAOA) and dopamine transporter (DAT) in SK-NF-I cells

	0 hr	3 hrs	6 hrs	24 hrs	48 hrs
**COMT**	1.00 ± 0.18	0.77 ± 0.12	0.95 ± 0.17	1.46 ± 0.23*	0.69 ± 0.08
**MAOA**	1.00 ± 0.11	1.06 ± 0.13	1.18 ± 0.33	1.88 ± 0.16**	0.86 ± 0.10
**DAT**	1.00 ± 0.24	0.78 ± 0.11	1.14 ± 0.15	1.46 ± 0.24	0.66 ± 0.08

Parallel cell cultures were incubated in 1% hypoxia for the indicated time periods. cDNA samples were prepared from three independent cultures and amplified in three parallel PCR reactions (N = 9). Relative expression levels were calculated as means and standard deviations of  values normalized to expression levels of the RPLP0 internal control gene. Data presented in the table were generated by dividing these normalized expression levels by that of the 0-hr control (*: p < 0.05; **: p < 0.01 and ***: p < 0.001).

### DRRF transcription is repressed by hypoxia

**Figure 6:**
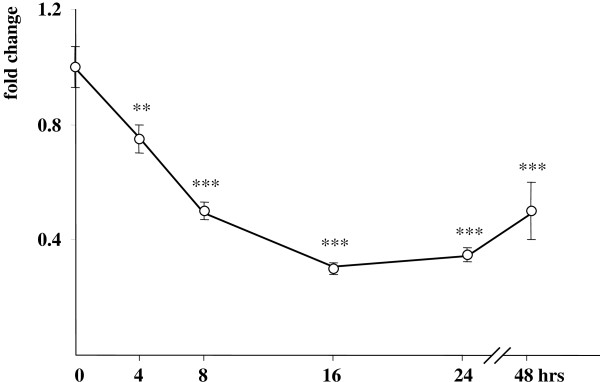
**DRRF transcript levels in SK-NF-I cells exposed to hypoxia**. Cell cultures were kept either in normoxia or in a 1% oxygen atmosphere for the indicated time periods. Cells were subsequently harvested, cDNA was synthesized and DRRF mRNA levels were measured using a specific Taqman probe. Expression levels were normalized to the RPLP0 internal control gene. All data are expressed as fold changes relative to levels measured in parallel normoxic samples. Data are means of three parallel determinations from three independent experiments (N = 9); bars represent standard deviations (*: p < 0.05; **: p < 0.01 and ***: p < 0.001).

DRRF (dopamine receptor regulating factor, Kruppel-like factor 16) is a zinc finger transcription factor that is considered a key regulator of post-synaptic dopamine receptors. DRRF has reportedly modulated DRD1, DRD2 and DRD3 promoter activities in a cell specific manner [29]. We were prompted to check whether activation of DRD3 and DRD4 promoters might be due to altered DRRF expression in hypoxia. It turned out that 1% hypoxia repressed DRRF levels in a time-dependent manner in SK-NF-I cells. Its concentration was the lowest after 16 hr hypoxia and then slightly elevated up to 48 hrs (Fig. 6). In conclusion, there seems to be an inverse correlation between DRD3/DRD4 and DRRF levels in the context of SK-NF-I neuroblastoma cells.

### No elevation of DRD3 and DRD4 protein levels by hypoxia

**Figure 7:**
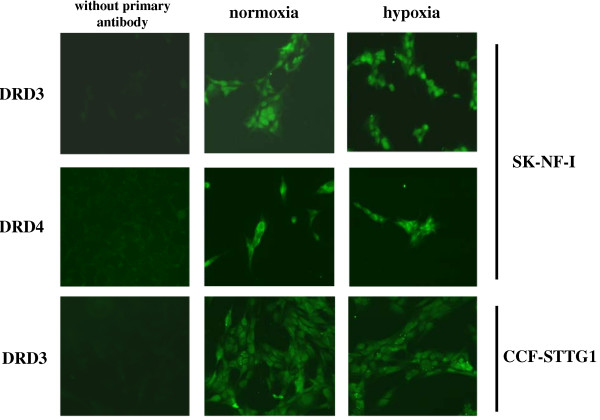
**No change in DRD3 and DRD4 protein levels upon hypoxia**. 48-hour hypoxic (1% O_2_) or control (21% O_2_) SK-NF-I and CCF-STTG1 cultures were immunostained with anti-DRD3 (1:50) or anti-DRD4 (1:250) antibodies, respectively. Representative images from three independent experiments (N = 3) are shown. The first column displays backgrounds obtained by omitting the primary antibody from the staining assays.

Having demonstrated the hypoxia sensitivity of the DRD3 and DRD4 genes, we aimed to examine whether elevated mRNA levels of both genes correlate well with protein expression. To this end, neuroblastoma and astrocytoma cells were cultured parallel for 48 hrs in normoxia or in 1% hypoxia, respectively, then fixed and immunostained with specific anti-DRD3 and anti-DRD4 antibodies. Cells were homogenously stained with marked cortical enrichment (Fig. 7), a pattern characteristic of membrane surface receptors [30]. We quantified staining intensities of corresponding normoxic and hypoxic samples by subjecting 500-500 cells to densitometry; however, no significant changes were revealed (data not shown). One can conclude that the transcriptional activation of DRD3 and DRD4 genes was not followed by elevation of their protein levels in these two cell lines.

## Discussion

Since we chose human neural tumour cell lines as experimental model systems, it was mandatory to verify that the hypoxic signalling pathway is intact and functional in these cells. Checking the mere expression of HIF-1α by western blot is, however, not sufficient to claim that SK-NF-I and CCF-STTG1 cells express a functional HIF-1α variant (Fig. 1). For instance, others found that the transcriptional activity of HIF-1 can be strongly impaired without simultaneous reduction in HIF-1α protein levels under certain conditions [31], and accumulation of non-functional HIF-1α has also been reported in normoxic cells [32]. Results presented in Fig. 3 clearly demonstrated that HIF-1α was highly functional in our experimental system as it transactivated an artificial hypoxia-responsive promoter construct several fold upon hypoxia. Importantly, the desferrioxamine treatment used as a positive control did not elicit a transactivation commeasurable with that of 1% hypoxia (5.5 fold versus 8.5 fold; Fig. 3, columns 2 and 3), although the expression patterns of HIF-1α in SK-NF-I cells were highly similar in both cases as evaluated by western blot (Fig. 1, upper panel). This marked difference might be attributed to the fact that HIF-1α is modified post-transcriptionally both by proline and asparagine hydroxylases in its oxygen-dependent degradation domain and in its C-terminal transactivation domain, respectively [33]. Proline hydroxylation destabilizes HIF-1α by facilitating its interaction with the von Hippel-Lindau protein, while asparagine hydroxylation by FIH-1 abrogates its interaction with the coactivator protein CBP/p300. No hydroxylation takes place in hypoxia, therefore HIF-1α is fully functional, while DFO treatment might not block fully asparagine hydroxylation, leading to the accumulation of HIF-1α with compromised transactivation potential.

In the RT-PCR assays we *ab ovo *excluded GAPDH, one of the most frequently employed internal control genes, due to its explicit hypoxia sensitivity [34,35], although early studies with hypoxia relied upon this conventional control gene [36]. In accordance with data of Zhong and Simons [34], β-actin did not prove to be a reliable internal control gene in the context of our cells too (Table 1).

Although the positive control gene VEGF was induced in both cell lines in hypoxia as expected, its expression patterns were slightly different (Figs. 4 and 5, dashed line). This observation might be related to the fact that CCF-STTG1 cells predominantly express the higher molecular weight variant of HIF-1α (Fig. 1) that might bind the VEGF promoter with slightly different affinity.

Regarding the hypoxic induction of the dopamine receptor genes, two important conclusions could be drawn.

First, the profound difference between the induction patterns of DRD3 and DRD4 and the direct HIF-1α target gene VEGF implies that the DRD3 and DRD4 promoters might not be activated directly by HIF-1α but other slow-reacting hypoxia-sensitive transcription factors might be involved in their transcriptional regulation (Figs. 4 and 5). Apart from the HIF family, hypoxia activates a cohort of other well-known transcription factors, such as NF-κB [37], AP-1 (activator protein 1) [38], the tumour suppressor p53 [39] and c-Myc [40], among others. These factors activate target promoters alone or in concert with HIF family members; moreover, they might modulate HIF-1α expression, eliciting a protracted transcriptional activation of HIF-1α target genes in hypoxia [41]. On the other hand, p53, c-Myc and NF-κB have been shown to induce microRNAs that may reflect another level of control of the hypoxic response [42]. Unfortunately, the promoters of DRD3 and DRD4 receptors have not yet been characterized thoroughly, and further investigations such as chromatin immunoprecipitation assays are needed to explore whether the above mentioned transactivators can really be recruited to these promoters *in vivo*. Interestingly, although DRD2 is a known target gene of the hypoxia-inducible NF-κB [43], we did not observe any hypoxia-related alterations in its expression in the context of our cell lines (Figs. 4 and 5). A similar contradiction has been revealed upon studying the expression pattern of DRRF in hypoxia. Although there are consensus binding sites for AP-1 in the DRRF promoter [44], repressed DRRF mRNA levels were detected upon hypoxia (Fig. 6). On the other hand, it is tempting to assume that DRRF might repress directly or indirectly the DRD3 and DRD4 promoters in cells as the expression patterns of these genes were inverse: DRRF levels were the lowest after 16–24 hr hypoxia when DRD3 and DRD4 mRNA concentrations started to elevate (Figs. 4 and 5).

Second, the observation that SK-NF-I cells preferentially expressed DRD4 while DRD3 was mostly activated in CCF-STTG1 cells might be due to different expression of critical, gene-specific transcription factors, or to epigenetic differences in chromatin structure or in hypoxia-responsive remodelling of chromatin (histone acetylation, methylation etc.). This issue is particularly interesting in the light of recent reports claiming that HIF-1α is capable of interacting both with histone acetyltransferases and deacetylases [45,46]. We currently try to address the epigenetic regulation of dopaminergic neurotransmission related genes by using specific modulators of chromatin modifying enzymes.

In spite of the profound transcriptional activation of DRD3 and DRD4 promoters in hypoxia, we could not detect elevated protein levels by immunostaining of either SK-NF-I or CCF-STTG1 cultures (Fig. 7). One can speculate that the duration of hypoxic treatment (48 hrs) might not have been enough for protein synthesis, although DRD3 mRNA levels were elevated already upon 24 hrs incubation under hypoxic conditions in astrocytoma cells (Fig. 5). On the other hand, it can be assumed that translation of these transcripts was strongly blocked by hypoxia. A large body of experimental evidence suggests that hypoxia can reduce cellular energy levels, leading to activation of the AMP-activated protein kinase (AMPK) that downregulates mammalian target of rapamycine (mTOR) activity, a critical stimulator of translation [47]. Moreover, hypoxia has been reported to activate PERK (PKR-like endoplasmic reticulum kinase) that inactivates the eIF2α translation initiation factor by phosphorylation [48]. However, particular mention must be made of the results of [49] who reported that 2-hr oxygen and glucose deprivation increases DRD2 and DRD3 protein expression in rat oligodendrocytes. Unfortunately, they did not study the effect of hypoxia alone on the expression of these receptors, therefore their results are not directly comparable with ours.

## Conclusion

In the present study we reported for the first time the hypoxia-induced transcriptional activation of the dopamine D3 and D4 receptor genes. However, the molecular mechanism of transactivation remains to be elucidated as our data indicate that these promoters might not be targeted directly by HIF-1α. Nevertheless, modulation of postsynaptic dopamine receptor genes by hypoxia might play a role both in the formation of dopaminergic circuitries in the developing brain and in the adaptation of neurons to post-ischemic conditions.

## Methods

### Plasmid constructions

The pSV40-5×HRE-luc-Kana reporter vector was constructed by subcloning the VspI-BamHI fragment of the pGL3-5×HRE-Control vector, bearing the SV40 promoter and the luciferase gene, into the VspI-BamHI site of the pEGFP-C2 vector. The pGL3-5×HRE-Control vector, containing five contiguous hypoxia responsive elements (5'-GATCTGAGACAGCACGTAGGGC-3') upstream of the luciferase reporter gene, was a generous gift from Dr. M. Geiszt (Institute of Physiology, Semmelweis University, Budapest, Hungary).

### Cell culture, treatments and transfections

The SK-NF-I human neuroblastoma cell line and the CCF-STTG1 human astrocytoma cell line were maintained in Dulbecco's modified Eagle's medium (DMEM) supplemented with 10% fetal bovine serum, 100 U/ml penicillin and 100 μg/ml streptomycin. Normoxic cultures as well as samples treated with the iron chelator desferrioxamine (DFO; 100 μM final concentration) were kept in 21% O_2_, 74% N_2 _and 5% CO_2 _in humidified atmosphere. Hypoxic samples were incubated in a humidified atmosphere of 1% O_2_, 94% N_2 _and 5% CO_2 _in a modular incubator chamber (Billups-Rothenberg, USA). All reagents were of analytical grade and obtained from Sigma-Aldrich Co. Cell viability exceeded 95% throughout all experiments as proven by the trypan blue exclusion test.

In reporter assays, 1.5 × 10^6 ^cells were transiently cotransfected with 0.3 μg pSV40-5×HRE-luc-Kana reporter plasmid and 0.1 μg pCMV-β-gal using the Lipofectamine reagent (Invitrogen). At 24 hrs after transfection the cells were subjected to hypoxia or incubated with DFO for 24 hrs, respectively, as indicated. Cells were extracted by three consecutive freeze-thaw cycles in Tris-HCl buffer (250 mM, pH 8.0), and luciferase and β-galactosidase activities were determined as reported earlier [50].

### HIF-1α immunoblotting

Cells were treated and washed as described above. Pellets were resuspended in a freshly prepared lysis buffer containing 50 mM Tris-HCl pH 7.6, 150 mM NaCl, 10% (V/V) glycerol, 2 mM DTT, 0.5% (V/V) NP-40, 5 mM EDTA, 1 mM Na-vanadate, 1 mM PMSF, 20 mM NaF, 10 mM benzamidine, 10 mM lactacystin (a proteasome inhibitor), supplemented with Complete Protease Inhibitor Cocktail (Roche). Cells were disrupted by sonication on ice with a Vibra-Cell device (Sonics & Materials, USA) at 20 kHz and 25 W output by 3 × 10 s pulses. Supernatants were clarified by centrifugation (14,000 g, 20 min, 4°C). The protein concentration of cleared supernatants was determined with the Bio-Rad D_C _Protein Assay kit. Samples were diluted to equal protein concentration and supplemented with equal volumes of 2× Laemmli buffer followed by heat denaturation (100°C, 5 min). Approximately 25 μg of total protein were resolved by SDS-PAGE on 8% gel slabs and subjected to western blotting as described in [51].

Membranes were immunoblotted with a polyclonal anti-human HIF-1α primary antibody at 1:2,000 dilution for 60 min and subsequently with a secondary anti-mouse antibody derived from goat at 1:4,000 dilution (60 min, room temperature). Immunocomplexes were visualized by the enhanced chemiluminescence reaction (Amersham Life Sciences). Three blots were made from 3 independent biological samples.

### Extraction of total RNA, cDNA synthesis and qRT-PCR assays

Total RNA was isolated by the RNeasy kit (Qiagen), according to the manufacturer's instructions. The quality of the preparation was checked by running an aliquot on ethidium bromide stained agarose gels.

cDNA was reverse transcribed with the High-Capacity cDNA Archive Kit (ABI). The reaction contained 0.2 μg total RNA, 5 U/μl MultiScribe™ Reverse Transcriptase and 1× relative concentration of Reverse Transcription Buffer, dNTPs and random primers in 50 μl final volume. The reaction was incubated at 25°C for 10 min and then at 37°C for 120 min.

Real-time PCR assays were performed in 25 μl final volume containing 5 μl cDNA, 1× ABI PCR master mix, gene-specific TaqMan^® ^primers and the gene-specific, FAM-labelled probe. Amplification and signal detection were performed using an ABI 7300 Real-Time PCR System (Applied Biosystems). Denaturation at 95°C, 10 min was followed by 40 thermocycles (95°C, 15 sec and 60°C, 1 min). Reactions were performed from three independent biological replicates in triplicate using RNase-free water as negative control. C_T_-values were set in the exponential range of the amplification plots using the 7300 System Sequence Detection Software 1.3. ΔΔC_T_-values corresponded to the difference between the C_T_-values of the genes examined and those of the RPLP0 calibrator (internal control) gene. Relative expression levels of genes were calculated and expressed as 2^-ΔΔCT^. To minimize the effect of pipetting errors, the TaqMan reaction mixture contained 6-carboxy-X-rhodamine (ROX) as a passive reference calibrator fluorescent dye.

The following TaqMan^® ^assays (Applied Biosystems) were used in this study: HGPRT (Hs99999909_m1); RPLP0 (Hs99999902_m1); HMBS (Hs00609297_m1); RPII (Hs00172187_m1); β-actin (Hs99999903_m1); VEGF (Hs00900058_m1); DRD2 (Hs01024460_m1); DRD3 (Hs00364455_m1); DRD4 (Hs00609526_m1); DRRF (Hs00259103_m1); COMT (Hs02511558_s1); MAOA (Hs00165140_m1); DAT (Hs00997371_m1).

### Immunostaining

2 × 10^5 ^cells were grown on coverslips for 2 days in 1% oxygen. Then the cells were washed thrice with PBS and fixed for 20 min with 4% formalin. After fixation the coverslips were washed thrice with PBS, permeabilized with 0.1% Triton-X100, washed with PBS and blocked in PBS containing 5% fetal calf serum (FCS) for 1.5 hrs. α-DRD3 (sc-9114, Santa Cruz Biotechnology) and α-DRD4 (AB1787P, Millipore Co.) antibodies were diluted 1:50 and 1:250, respectively, in 1% FCS/PBS and applied to the cells overnight. After washing with FCS/PBS, the cells were incubated with diluted (1:1000), Alexa488-conjugated anti-rabbit secondary antibodies for 60 min, washed with PBS and mounted on glass slides with Mowiol (Polysciences). Fluorescence images were obtained and photographed in a Leitz Dialux 20 EB microscope equipped with epifluorescence optics. Staining intensities were quantitated by the ImageJ image processing software.

### Statistical analysis

Statistical analysis was performed with one-way analysis of variance (ANOVA) followed by the Tukey-Kramer Multiple Comparison Test (GraphPad InStat software).

## Authors' contributions

MB carried out most of the experimental work, EK and VM performed some of the western blotting and real-time PCR assays. MS participated in the design of the study and helped to evaluate the results. GK coordinated the study and wrote the manuscript. All authors read and approved the manuscript.
